# Intraoperative resection control using arterial spin labeling — Proof of concept, reproducibility of data and initial results

**DOI:** 10.1016/j.nicl.2017.04.021

**Published:** 2017-04-25

**Authors:** Thomas Lindner, Hajrullah Ahmeti, Isabel Lübbing, Michael Helle, Olav Jansen, Michael Synowitz, Stephan Ulmer

**Affiliations:** a Department of Radiology and Neuroradiology, University Hospital Schleswig-Holstein Campus Kiel, Arnold-Heller-Str. 3, 24105 Kiel, Germany; b Clinic for Neurosurgery, University Hospital Schleswig-Holstein Campus Kiel, Arnold-Heller-Str. 3, 24105 Kiel, Germany; c Philips GmbH Innovative Technologies, Research Laboratories, Röntgenstraße 24-26, 22335 Hamburg, Germany; d Medizinisch Radiologisches Institut, Bahnhofplatz 3, 8001 Zurich, Switzerland

**Keywords:** Arterial spin labeling, ASL, Intraoperative, Monitoring, Perfusion

## Abstract

**Objectives:**

Intraoperative magnetic resonance imaging is a unique tool for visualizing structures during resection and/or for updating any kind of neuronavigation that might be hampered as a result of brain shift during surgery. Advanced MRI techniques such as perfusion-weighted imaging have already proven to be important in the initial diagnosis preoperatively, but can also help to differentiate between tumor and surgically induced changes intraoperatively. Commonly used methods to visualize brain perfusion include contrast agent administration and are therefore somewhat limited. One method that uses blood as an internal contrast medium is arterial spin labeling (ASL), which might represent an attractive alternative.

**Materials and methods:**

Ten healthy volunteers were examined using three different scanners and coils within 1 h (3T Achieva MRI using 32-channel head coil, 1.5T Achieva MRI using a 6-channel head coil, 1.5 Intera Scanner using 2 surface coils, Philips, Best, The Netherlands) and quantitative CBF values were calculated and compared between the different setups. Additionally, in eight patients with glioblastoma multiforme, ASL was used pre-, intra-, and postoperatively to define tumor tissue and the extent of resection in comparison to structural imaging.

**Results:**

A high correlation (r = 0.91–0.96) was found between MRI scanners and coils used. ASL was as reliable as conventional MR imaging if complete resection was already achieved, but additionally provided valuable information regarding residual tumor tissue in one patient.

**Conclusions:**

Intraoperative arterial spin-labeling is a feasible, reproducible, and reliable tool to map CBF in brain tumors and seems to give beneficial information compared to conventional intraoperative MR imaging in partial resection.

## Highlights

• The use of arterial spin labeling intraoperatively during neurosurgical interventions is presented.
• Reproducibility and comparability using various field strengths were successfully proven in a volunteer cohort.
• Detection of residual tumor in patients was compared to contrast-enhanced imaging.
• Residual tumor could be identified with a high accuracy.

## 1 Introduction

Magnetic resonance imaging (MRI) represents the gold standard for perioperative imaging of brain tumors as it allows for the submillimeter acquisition of fine anatomical and pathological structures with excellent soft-tissue contrast. In brain tumor surgery, the therapeutic aim is complete resection of a lesion while simultaneously preventing damage to “eloquent” brain areas (e.g., the speech areas). The extent of resection comprises one of the prognostic factors for progression-free and overall survival alike (Keles et al., 1999, Sanai and Berger, 2009). Intraoperatively, brain shift after craniotomy or during resection of the lesion can unpredictably invalidate the preoperatively acquired data (Nabavi et al., 2001). To correct for this brain shift, intraoperative imaging was introduced (Black et al., 1997). Intraoperative MRI examinations require particular equipment (e.g., surface coils) and patient positioning is limited. Air-fluid borders or an air-filled resection cavity may affect image quality in some sequences or produce distinctive susceptibility artifacts, which may preclude identification of the resection cavity rim or residual tumor tissue. However, surgery itself temporarily disrupts the blood-brain barrier intraoperatively, causing contrast enhancement at the rim of the resection cavity, which possibly cannot be distinguished from residual tumor using conventional sequences only (Ulmer et al., 2010, Knauth et al., 1999a).

In addition to structural (anatomical) imaging, obtaining physiological information (i.e., perfusion MRI) is helpful for differential diagnosis as well as for postoperative follow-up imaging to depict recurrent disease or to distinguish it from postoperative changes. Since being introduced, intraoperative perfusion imaging has proven to be a reliable method of identifying residual tumor during surgery and distinguishing it from surgically induced contrast enhancement that potentially can lead to misinterpretation of these images (Knauth et al., 1999b, Ulmer et al., 2009, Özduman et al., 2014). However, dynamic susceptibility-weighted MRI (DSC-MRI) or dynamic contrast-enhanced MRI (DCE-MRI) requires intravenous administration of a contrast agent (CA). The use of such agents represents a disadvantage of these methods as it limits the number of times the measurements can be repeated. Furthermore, it can be problematic in patients in whom renal function is impaired, potentially leading to nephrogenic system fibrosis (NSF) (Kaewlai and Abdujeh, 2012). Additionally, recent findings suggest that MRI contrast agents become dechelated, causing permanent accumulation of by-products in the brain parenchyma (Errante et al., 2014, Kanda et al., 2015).

One method of obtaining perfusion contrast without the need for an external contrast agent is arterial spin labeling (ASL). Here, an endogenous contrast agent is created by manipulating the magnetic state of inflowing blood, whereby a label (inversion) and a control (no inversion) image are acquired which are subsequently subtracted to obtain cerebral blood flow (CBF) weighted images (Alsop et al., 2015, Wong, 2014, Buxton et al., 1998). ASL is attractive as not only is a CA application not required, but CBF can also be quantified in absolute values (ml/min/100 g brain tissue) (Wong, 2014, Buxton et al., 1998). Furthermore, while ASL benefits from higher magnetic field strengths (3T), it can also be reliably performed on 1.5T scanners (Alsop et al., 2015, Ostergaard et al., 1996). Currently, the recommended method of performing non-contrast-enhanced perfusion imaging using ASL is pseudo-continuous ASL (pCASL) (Alsop et al., 2015). In this method, inversion of inflowing blood is achieved by applying short, consecutive RF pulses in conjunction with pulsed gradient fields in a tagging plane placed across the arteries in the neck. After tagging, a certain delay time needs to be awaited, which is denoted as postlabeling delay (PLD). At the time of acquisition, all blood is ideally delivered to the parenchyma. Owing to the low signal that is obtained in each acquisition, the experiment needs to be repeated several times, giving acquisition times of approximately 5 min in total (Alsop et al., 2015).

The first aim of this study was to evaluate the reproducibility of ASL using different scanners, scanning equipment, and field strengths to investigate whether the results of quantitative CBF measurements (of pre-, intra-, and postoperative imaging) match. These measurements were performed in a volunteer cohort without any history of intracranial disease. In addition to investigating the reliability of ASL, the clinical value of the technique for intra- and postoperative resection control was assessed in a small series of patients suffering from glioblastoma multiforme (GBM) in direct comparison to anatomical imaging.

## 2 Materials and methods

Written informed consent was obtained prior to the study, which was approved by the local ethical committee (IRB).

### 2.1 Magnetic resonance imaging

MRI in this study was performed on three different scanners (Philips Healthcare, Best, The Netherlands), including a 3T (Achieva series) scanner equipped with a 32-channel receive head coil, a 1.5T (Achieva series) scanner with a 6-channel head coil, and another 1.5T scanner (Intera series) equipped with two one-channel circular surface coils located in the operating room (OR). The imaging parameters for each scanner are summarized in Table 1.

**Table 1:** Acquisition parameters for the ASL scan and the T2-weighted anatomical reference scan (used for cortical segmentation in the volunteers).

Parameter	Pseudo-continuous arterial spin labeling scan	T2-weighted scan
Scan technique	Echo-planar-imaging	Turbo-spin-echo
TR/TE (ms)	2616/13	1858/80 (3524/110 at 1.5T)
EPI/TSE factor	39 (33 at 1.5T)	19 (23 at 1.5T)
In-plane voxel size (mm)	3.6 × 3.5	2.7 × 2.7
SENSE factor	2	–
Slice thickness (mm)/number of slices	5/16	5/16
Field-of-view (mm)	240 × 240 × 95	240 × 240 × 95
Readout flip angle	90°	90°
Labeling duration/post labeling delay (ms)	1800/1800	–
Number of label and control pairs (3T/1.5T Rad/1.5T OR)	20/30/40	–

### 2.2 Volunteer study

Ten healthy volunteers (6 female and 4 male, age = 31.4 ± 16.2 years; mean ± standard deviation) were included in this study. These patients were recruited from the neurosurgery department after spinal surgery. Exclusion criteria were any history of intracranial conditions and general MRI incompatibility, e.g., due to cardiac pace makers.

Each volunteer was examined in each of these MRI scanners within 1 h to minimize any fluctuations in CBF, e.g., caused by varying blood pressure. The data obtained from this study were acquired to ensure comparability between the different scanners. The acquisition protocol included a T2-weighted sequence serving as an anatomical reference and for subsequent gray matter segmentation. Additionally, a pseudo-continuous arterial spin labeling (pCASL) scan with echo-planar readout (EPI) was performed (Alsop et al., 2015). Acquisition details are presented in Table 1.

### 2.3 Patient study

To show the potential of ASL in the intraoperative setting, eight patients suffering from GBM (5 male, 3 female, age = 59.2 years ± 22.3; mean ± standard deviation) were included. Of the eight patients, four had recurrent tumor mass (RT) and were referred to surgery after undergoing an MRI examination. These patients suffering from primary tumor (P) were admitted to MRI based on their clinical presentation, e.g., nausea, headache, focal seizures, or speech disturbance or a combination of these symptoms. One patient was referred to surgery after undergoing neoadjuvant chemotherapy (P(CTx)). All patients had visible tumor mass in both ASL and anatomical imaging prior to surgery. Exclusion criteria were general incompatibility with MRI scanning. All patients were scanned peri-, intra-, and postoperatively.

### 2.4 Preoperative imaging

MRI was performed in all patients for the purpose of neuronavigation (Brainlab VectorVision Sky ver. 6.01, Munich, Germany) the day before surgery. At this examination a contrast-enhanced T1-weighted dataset (MPRAGE) is acquired after attaching five fiducial markers to the patient's head to enable image-to-patient registration in the OR. In five patients, ASL was performed prior to contrast administration in this scan session on either the 3T or the 1.5T of the radiology department. The remaining three patients underwent preoperative ASL in the OR just prior to surgery. A summary of the scanners used is listed in the Appendix A, Table 2.

### 2.5 Intraoperative setting

In the OR, conventional microsurgical equipment is used for tumor surgery, which needs to be placed and remain outside the 5-G line, including the intraoperative microscope and the anesthetic equipment. Anesthesia was induced intravenously in all patient cases using 3 mg/kg/h of propofol and 0.3 μg/kg/min remifentanil after initial administration of a bolus of both drugs that was adapted to the patients' bodyweight. The OR table and the MRI can be connected using a modified rotating table (DIAGNOST 5 Syncra Tilt Patient Support, Philips Medical System, Best, The Netherlands) that can be attached to both the scanner and the OR table socket without repositioning the patient. The head of the patient is fixed using a carbon-fiber Mayfield head-holder (ProMedics, Dusseldorf, Germany). After anesthesia and positioning of the patient, image-to-patient registration was performed based on the postcontrast MPRAGE dataset. Neuronavigation was guided using a ceiling-mounted infrared tracker. MRI was performed intraoperatively either if the surgeon had estimated complete removal of the lesion or if the surgeon decided that further resection was not safe (e.g., due to adjacent eloquent structures).

For imaging the resection cavity was flushed using saline and filled with sterile swabs. The whole cavity was then draped with sterile covers. Circular coils wrapped into sterile draping were positioned below and above the patients' head (anterior and posterior). This routinely performed intraoperative imaging protocol includes an axial T2-weighted sequence and sagittal and coronal T1-weighted images. The T1-weigthed images were acquired prior to and after administration of CA. Owing to the limitations of ASL being performed after CA administration, ASL was performed beforehand. Total surgery time including MRI was 4:43 ± 1:04 (mean ± standard deviation) h. Detailed durations for surgery can be found in Appendix A, Table 2.

### 2.6 Postoperative management

After surgery, all patients were transferred to the intensive care unit. Postoperative MRI is routinely performed within 24 h after surgery to judge the extent of the resection and to detect any unforeseen complications. Imaging was performed again on the 1.5T or 3T scanner in the radiology department and the protocol included diffusion-weighted imaging, an axial T2 image, and the same T1-weighted images as during surgery with and without CA. ASL was again performed prior to administering CA.

### 2.7 Data processing

All data were exported and postprocessed offline on a personal computer running Matlab R2015a (The Mathworks, Natick, MA). In the volunteer study, the anatomical images were segmented for gray matter using statistical parametric mapping (SPM, version 12, The Wellcome Department of Cognitive Neurology, Institute of Neurology, University College London) to evaluate mean gray matter perfusion only. The resulting binary mask after segmentation consisting of gray matter only was applied to the ASL images. After applying the mask, the average of whole-brain gray matter CBF could be compared between the scanners. To quantify CBF values in ml/100 g/min, the algorithm recommended by the ISMRM perfusion study group and ASL consortium in dementia was employed (Alsop et al., 2015):

$$\mathrm{CBF}\;\left[\mathrm{ml}/\min/100g\right]=\frac{6000^{*}\lambda^{*}{\left({\mathrm{SI}}_{\mathrm{subtracted}}\right)}^{*}e^{\frac{\mathrm{PLD}}{{T1}_{\mathrm{blood}}}}}{2^{*}\alpha^{*}{{T1}_{\mathrm{blood}}}^{*}{M_{0}}^{*}\left(1-e^{-\frac{\tau }{{T1}_{\mathrm{blood}}}}\right)}$$

where SI_subtracted_ is the signal intensity of the subtracted label and control images, λ is the blood-brain partition coefficient (0.9 mL/g), PLD is the postlabeling delay (1800 ms), T1_blood_ is the T1 relaxation constant of blood (1650 ms at 3T and 1350 ms at 1.5T), M_0_ is the signal intensity of the M_0_ image, and τ is the labeling duration (1800 ms).

In patients, ASL quantification was performed without segmentation to avoid removing (parts of) the tumor and images were not registered between the scan sessions. In the present study, the ASL images were not included intraoperatively for judging the completeness of tumor removal, but processed off-line after surgery. However, the data can be easily post-processed in real-time intraoperatively to already obtain the information during the procedure.

### 2.8 Data analysis

Quantitative CBF values obtained in each volunteer at each scanner were compared by applying Pearson's correlation. High correlation coefficients between the different settings were a prerequisite for using ASL intraoperatively and exchanging scanners preoperatively. ASL was additionally used to assist judgment of complete removal. To do so, two independent reviewers blinded to any intraoperative information read the images: one read anatomical images only, the other ASL data only to avoid bias through tumor sizes and locations by re-evaluating images. Both readers had several years of experience reading these images. To assess the interrater reliability, the intraclass correlation coefficient (ICC) was calculated.

## 3 Results

### 3.1 Volunteer study

**Fig. 1:**
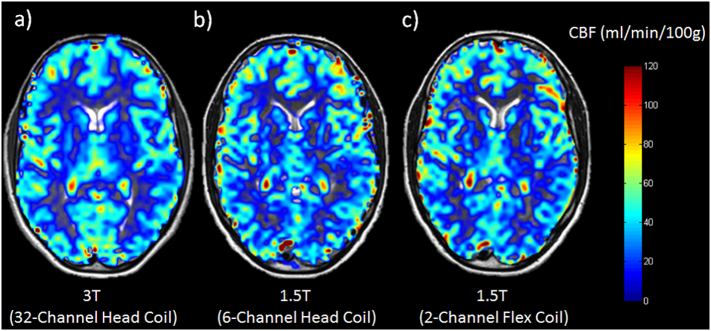
Representative CBF maps of one healthy volunteer who was scanned on the 3T (a) and the 1.5T in the radiology department (b) and the 1.5T in the OR (c). Subjective image quality appears best on the 3T (a), but the individual gyri can be delineated on the 1.5T scanners as well.

**Fig. 2:**
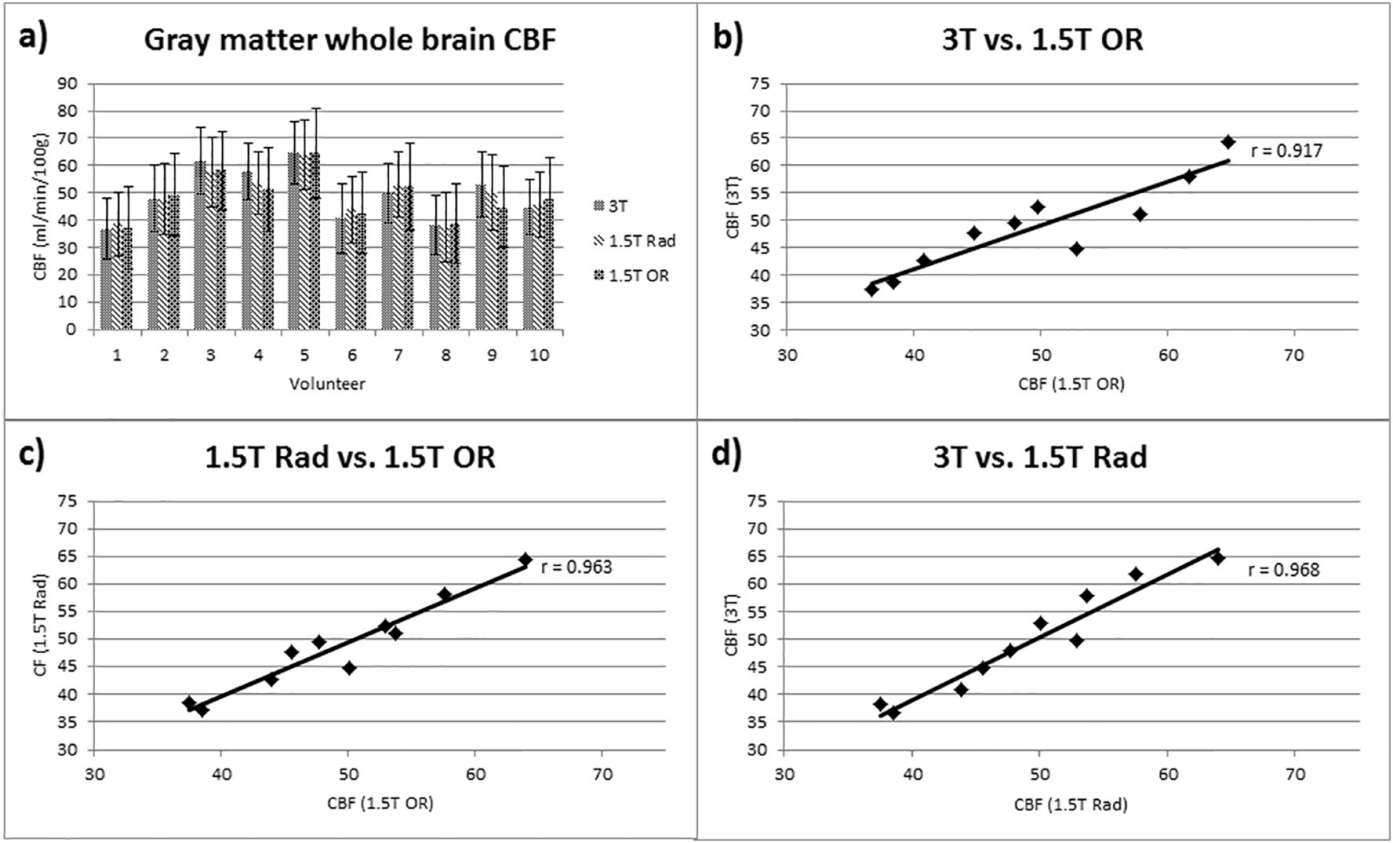
Correlation coefficients comparing the results from the 3T scanner with those of the 1.5T in the radiology (Rad) department (a), the 3T scanner with the intraoperative (OR) scanner (b), and the two 1.5T scanners (c). Mean results from the CBF measurements of all volunteers are shown in (d). The CBF values yielded results with some differences but correlation between the scanners was high. Higher standard deviations were found in the 1.5T scanners. Note that the interindividual deviation in CBF values is rather high throughout the volunteer cohort; yet, between the scanners, only minor differences can be found.

**Fig. 3:**
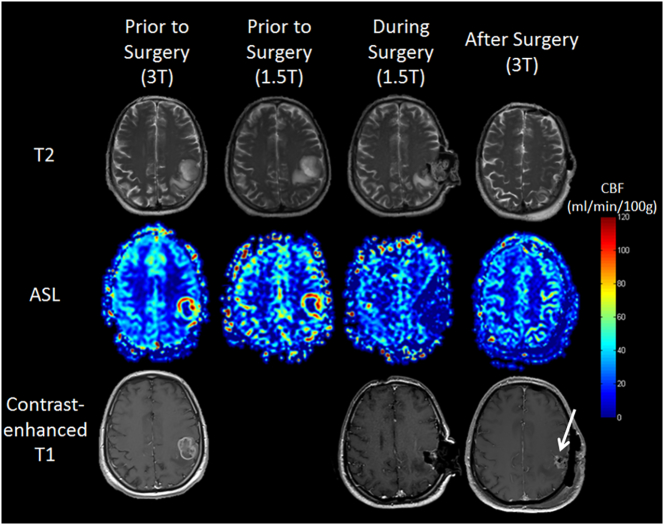
Example of a total resection (patient No. 1, Table 2). Images were acquired on the 3T and 1.5T scanners preoperatively (first and second left column), intraoperatively on the intraoperative scanner (third column), and postoperatively again on the 3T (right column). The contrast-enhanced T1-weighted images are shown in the bottom row. The ASL scans depicted the total resection in terms of no elevated CBF during and after surgery. Note that T1 weighted MR imaging “suggests” residual tumor tissue (arrow), which turned out to be an adjacent artery after including all postoperative imaging sequences.

Images were successfully acquired in all volunteers. Image quality and maps of quantitative CBF values present comparable results across the different scanners. An example of the images of all three scanners is shown for one representative volunteer in Fig. 1. The CBF values were within the range of previously published data (Petersen et al., 2010, Chen et al., 2011, Gevers et al., 2011). The mean (± standard deviation) of CBF values were 49.53 (± 9.72) for 3T, 49.15 (± 8.28) for 1.5T MRI, and 48.65 ± (8.43) ml/min/100 g for 1.5T MRI in the OR (surface coils); however, rather high interindividual deviations in CBF values were found within the volunteer cohort (Fig. 2). Quantitative values for each volunteer correlated very highly between settings (r_(3T vs 1.5T)_ = 0.968, r_(1.5T vs 1.5Tintraop)_ = 0.963, r_(3T vs 1.5Tintraop)_ = 0.917, Fig. 3). The CBF values measured for each individual volunteer can be found in Table 1 of the Appendix A.

### 3.2 Patient study

Among the eight patients undergoing brain tumor resection, five had residual tumor mass intraoperatively. Of these, one resection could be continued, leaving four patients with a visible postoperative tumor mass. In the remaining three, complete resection was already achieved during surgery (Table 2). The ICC for the data acquired intraoperatively was: 0.873 and for the postoperative data: 0.750.

**Table 2:** Intra- and postoperative evaluation of residual tumor mass using arterial spin labeling (ASL) and anatomical imaging.

Patient data		Arterial spin labeling		Anatomical imaging	
No.	Diagnosis	Intraoperative	Postoperative	Intraoperative	Postoperative
1^a^	RT	0	0	0	1 (0)^a^
2	RT	1	1	1	1
3	RT	0	0	0	0
4	P	1	1	1	1
5	P (CTx)	0	0	0	0
6^b^	P	1	1^b^	1	0^b^
7	RT	1	0	1	0
8	P	1	1	1	1

In this rating, 0 means no visible residual and 1 means visible residual tumor. The ICC for the intraoperative acquired data was: 0.873 and for the postoperative data: 0.750. RT: recurrent tumor; P: primary tumor; CTx: (neoadjuvant) chemotherapy.

a At first a structure was misinterpreted as residual tumor mass but later amended to being a partial volume effect of an artery (see also Fig. 2).

b In this case residual tumor was visible intraoperatively in both ASL and anatomical imaging, postoperatively only in the ASL. The contrast-enhanced T1 images needed to be reevaluated and it was concluded that there is still residual tumor mass (see also Fig. 3).

In patient No. 1 total resection could be achieved and is shown in Fig. 3. Initially, residual tumor was assumed based on T1-weighted images only, but proved to be a partial volume effect of an adjacent artery including all MR sequences postoperatively. Using ASL, complete removal was already determined.

**Fig. 4:**
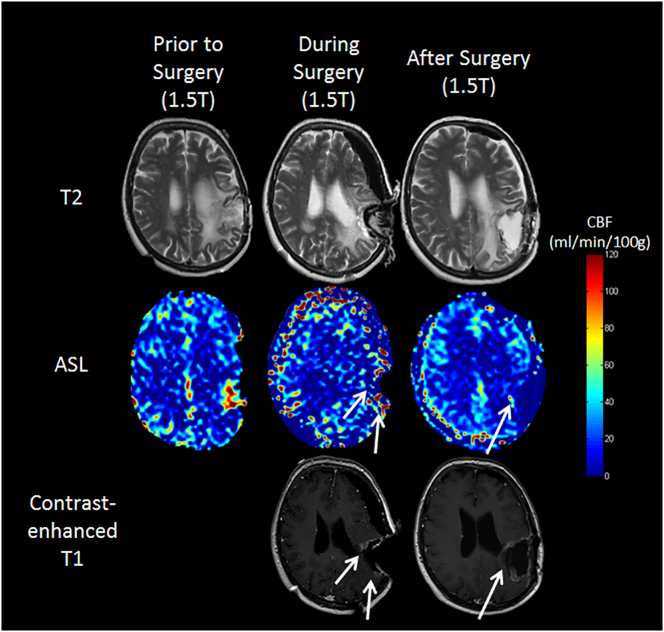
Images of one GBM patient (No. 6), whose tumor could not be resected in total (Table 2). Note that this patient shows rather strong brain shift, making interpretation of both ASL and anatomical imaging more difficult. The patient presented with complete preoperative MR imaging; thus, our data were acquired on 1.5T (pre- and intraoperatively in the OR) and according to the protocol postoperatively. In the preoperative scan, the area of elevated CBF can be clearly seen in the left parietal lobe (left column, middle). In both the ASL and structural imaging, residual tumor mass could be depicted during surgery (middle column, arrows). In the postoperative resection control (right column) the residual tumor mass is only seen as an area of elevated CBF in the ASL scan. On the contrast-enhanced T1-weighted images the residual tumor is not as prominent as any surgically induced changes and was overlooked at first in the blinded review.

Assessment for one patient (No. 6) in whom resection was incomplete was inconclusive between the readers and is presented in Fig. 4. Elevated perfusion was depicted in the ASL scan, indicating residual tumor which was not obvious solely on the contrast-enhanced T1-weighted images either intra- or postoperatively. As the results were inconclusive in this case, the patient was referred again to MR imaging. Additional scanning, including other sequences used postoperatively, revealed residual tumor mass that had been overlooked by only using contrast-enhanced T1-weighted images.

## 4 Discussion

Intraoperative MRI has been introduced to compensate for brain shift during resection of a lesion, but also to already control the extent of the resection intraoperatively while the skull is still open (Knauth et al., 1999b). In addition to brain shift, however, surgically induced temporary disruption of the blood-brain barrier could hamper interpretation of image data acquired intraoperatively. Any temporary intraoperative contrast enhancement could mislead the radiologist and surgeon (Black et al., 1997, Knauth et al., 1999a, Knauth et al., 1999b, Ulmer et al., 2009). To compensate for these constraints, DSC- and DCE-MRI have been used intraoperatively to help distinguish residual tumor from this surgically induced disruption of the blood-brain barrier (Knauth et al., 1999b, Ulmer et al., 2009, Özduman et al., 2014). For these techniques, CA must be administered intravenously (Korfiatis and Erickson, 2014). Intraoperative ASL was described recently in a small patient group under general anesthesia (Venkatraghavan et al., 2016). However, its use intraoperatively to depict (residual) brain tumor tissue has not been performed to date.

In the present study, we could demonstrate that ASL imaging produces robust, quantitative, interchangeable, and reproducible results in healthy volunteers using three different MRI scanners at different field strengths and using different types of receive coils. Intraoperatively, ASL can furthermore reliably depict residual tumor in glioblastoma patients. In addition, it seems to be even superior to conventional anatomical MR imaging intra- and postoperatively in this perspective, as shown in two of our cases. As surgically induced changes might be depicted in T1-weighted images both intra- and postoperatively, quantitative results from ASL are completely independent of these artifacts and enable a definite judgment.

Owing to the low signal changes in ASL between labeled and unlabeled images (even using 3T MRI scanners), the sequence must be repeated multiple times, even more so when using a lower field strength and/or with the use of one-channel circular coils (i.e., as available in the OR). Thus, the procedure takes more time than CA-based perfusion imaging techniques. Compared to DSC- or DCE-MRI, ASL has the advantage that adjacent blood vessels do not influence the results/perfusion maps, which is based on the assumption that all blood has been delivered to the tissue when calculating the final CBF values. The postlabeling delay (time between labeling and readout) is set to be longer than the arterial transit time; therefore, all blood has entered the tissue and no blood remains visible in large vessels (Alsop et al., 2015). However, due to pathological changes and also to anesthesia, blood flow velocities might slow down and blood potentially remaining inside the vessels can be misinterpreted as areas of elevated perfusion (overshoot) (Yoo et al., 2015).

ASL has the major advantage of being completely noninvasive. This becomes especially important when image acquisition needs to be repeated in cases of corrupted image acquisition data, which can occur more frequently in an intraoperative setting as the available equipment as well as the options for positioning the patients' heads and the available coils are limited. Movement artifacts constitute one major limitation of ASL in general, limiting the application to scan times of approximately 5 min or less. In the intraoperative setting, however, patient movement is restricted due to head fixation in a specially designed Mayfield holder and/or to anesthesia, making it possible to perform more acquisitions, thereby increasing the signal-to-noise ratio of the resulting images. Furthermore, repeated acquisitions are less of a concern in ASL imaging since, as opposed to CA-based perfusion techniques, no adverse effects of repeated imaging should be expected because repeated injection of contrast agents can be avoided (Kaewlai and Abdujeh, 2012, Errante et al., 2014, Kanda et al., 2015).

One limitation of this study is the low number of included patients, rendering it more difficult to interpret the clinical results. However, as the results appear conclusive between the two readers, the potential of using ASL in the intraoperative setting appears attractive for future studies in a larger patient cohort. Furthermore, as the patient group is heterogeneous (both primary and recurrent tumors), these two main groups should be investigated separately in future studies.

Another limitation is that CBF was not measured in low perfused tissue (i.e. white matter). Following, the reproducibility of the results could be inferior to the results obtained in this study for both gray matter and high-grade gliomas.

One major technical concern of intraoperative scanning per se is the air-fluid level, potentially distorting the images (susceptibility artifacts). However, this was negligible using EPI in DSC-MRI in phantom and in vivo studies performed previously (Ulmer et al., 2009) and again in the present study, underlining that EPI is a robust sequence for intraoperative MR imaging in our setting.

One problem regarding reproducibility in general is that pre- and postoperative MRI examinations are often conducted using different field strengths (as also employed in this study), and sometimes even on scanners from different manufacturers, making it difficult to accurately compare the imaging data obtained. This technical variety can be compensated by using ASL and creating the quantitative measurement of CBF (in ml/min/100 g brain tissue), as this value is – as presented both in previous studies and in this one – independent of the scanner hardware used (Mutsaerts et al., 2015).

## Conflict of interest

One of the authors (M.H.) is employed at Philips Research Laboratories, Hamburg, Germany.

## Figures and Tables

**Table 1:** CBF values (in ml/min/100 g) of the whole-brain gray matter for each volunteer on all three scanners with standard deviation.

Volunteer	CBF 3T	Std. dev.	CBF 1.5T Rad	Std. dev.	CBF 1.5T OR	Std. dev.
1	36.71	11.15	38.53	11.49	37.25	15.00
2	47.91	12.05	47.66	13.05	49.4	15.07
3	61.69	12.32	57.55	12.50	58.08	14.31
4	57.77	10.44	53.73	11.31	51.18	15.1
5	64.75	11.38	63.95	12.61	64.39	16.23
6	40.77	12.66	43.91	12.22	42.67	14.91
7	49.77	10.93	52.92	11.93	52.45	15.84
8	38.38	10.81	37.5	12.59	38.63	14.54
9	52.83	11.92	50.11	13.95	44.76	14.85
10	44.69	10.04	45.58	11.92	47.71	15.21
Mean	49.53	9.72	49.15	8.28	48.56	8.43

**Table 2:** List of the devices used for each patient for peri- and postoperative scanning alongside the duration of surgery of each patient.

Patient	Perioperative	Postoperative	Duration of surgery (hours:minutes)
1	3T	3T	04:49
2	1.5T Rad	1.5T Rad	05:23
3	1.5T NCH	1.5T Rad	04:51
4	1.5T NCH	1.5T Rad	06:16
5	3T	3T	02:35
6	1.5T NCH	1.5T Rad	04:19
7	1.5T Rad	1.5T Rad	05:13
8	1.5T Rad	1.5T Rad	04:21

**Table 3:** Representative example of the measured g-factors of one healthy volunteer in each setting showing a reduction of SNR from the 3T to the 1.5T Rad scanner and the intraoperative scanner.

3T	1.5T Rad	1.5T OR
1.12 ± 0.11	1.01 ± 0.12	0.97 ± 0.09

**Table 4:** Grading of image quality by two readers of the unsubtracted EPI images of the ASL datasets. The rating scale was: (1) excellent image quality without or very little distortion; (2) good image quality with minor distortions; (3) fair image quality with distortions, but usable for diagnosis; and (4) poor image quality, not possible to be used for interpretation. The ICC between the two readers was calculated as 0.676.

Patient	Reader 1	Reader 2
1	1	1
2	1	2
3	2	1
4	3	2
5	1	1
6	2	2
7	2	2
8	1	1
